# Gender based survival prediction models for heart failure patients: A case study in Pakistan

**DOI:** 10.1371/journal.pone.0210602

**Published:** 2019-02-19

**Authors:** Faisal Maqbool Zahid, Shakeela Ramzan, Shahla Faisal, Ijaz Hussain

**Affiliations:** 1 Department of Statistics / Government College University, Faisalabad, Pakistan; 2 Faisalabad Medical University, Allied Hospital, Faisalabad, Pakistan; 3 Department of Statistics / Quaid-i-Azam University, Islamabad, Pakistan; The University of Warwick, UNITED KINGDOM

## Abstract

**Objectives:**

The objective of this study was to build and assess the performance of survival prediction models using the gender-specific informative risk factors for patients with left ventricular systolic dysfunction.

**Methods:**

A lasso approach was used to decide the informative predictors for building semi-parametric proportional hazards Cox model. Separate models were built for all patients [N = 299], male patients [N_male_ = 194 (64.88%)], and female patients [N_female_ = 105 (35.12%)], to observe the risk factors associated with the individual’s risk of death. The likelihood- ratio test was used to test the goodness of fit of the selected model, and the C-index was used to assess the predictive performance of the selected model(s) with respect to the overall model with all observed risk factors.

**Results:**

The survival prediction model for females is notably different from that for males. For males, smoking, diabetes, and anaemia, whereas for females, ejection fraction, sodium, and platelets count are non-informative with zero regression coefficients. The goodness of fit of the selected models with respect to the general model with all observed risk factors is tested using the likelihood-ratio test. The results are in favor of the selected models with p-values 0.51,0.61, and 0.70 for all patients, male patients, and female patients, respectively. The same values of C-index for the full model and the selected models for overall data, for males, and for females (0.72, 0.73, and 0.77 for overall data, male data, and female data, respectively) indicate that the selected models are as good as the corresponding overall models regarding their predictive performance.

**Conclusion:**

There is a substantial difference in the survival prediction models for heart failure (HF) of male and female patients in this study. More studies are needed in Pakistan for confirming this striking male-female difference regarding the potential risk factors to predict survival with heart failure.

## 1 Introduction

Cardiovascular Disease (CVD) is the most fatal disease in the world. According to World Health Organization (WHO), 31% of all deaths around the globe are due to CVD [1]. Around 80-86% of deaths due to CVD in the world occur in low and middle-income countries [2]. In 2015, the estimated number of deaths due to CVD were 17.7 million, of which 7.4 million were due to the coronary heart disease, and 6.7 million were due to the stroke. Pakistan is a part of Eastern Mediterranean Region, and according to an estimate of WHO, 54% of deaths from noncommunicable diseases in this region are due to the cardiovascular problem [3].

Heart failure (HF) refers to the condition where the heart cannot pump blood properly to fulfill the need of the body. It is a serious problem with no cure in general. The incapacitated heart pumping causes fatigue and breathing problems. The conditions like coronary artery disease, hypertension, cardiomyopathy, congenital heart defects, and heart arrhythmia can cause heart failure. There are four types of HF: left-sided HF, right-sided HF, systolic HF, and diastolic HF. The systolic HF occurs when the heart does not pump vigorously to push enough blood into the body due to abnormal contraction of the ventricle. The ventricular systolic dysfunction is more common in males, whereas females are more associated with preserved left ventricular function [4].

In the literature, different findings are available for the gender-based risk factors associated with mortality of HF patients (see, for example, [5, 6]). However, the studies to compare survival rates of male and female HF patients are scarce [7]. It remains still unclear whether male and female have similar survival or not? It has been reported in some studies that female HF patients are more fragile than males, for example, [8] and [9]. Women, especially with increasing age, have more problem of Congestive HF with preserved systolic function than men [10]. In contrast, some studies conclude that women with HF are better survivors than men [11–13]. The association of survival with anemia and left ventricular EF for the patients with acute decompensated HF was investigated by Kajimoto et al. [14], and a significant difference was found between men and women. In the Western world, the prevalence of HF is higher in men than in women above 40 years, but situation is reversed after the age of 80 years [15]. Unfortunately, there are no reliable estimates of incidence and prevalence of HF available for south Asia region. McKeigue et al. [16] reported coronary heart disease rates from several parts of the world, and these rates are unusually high in the people originating from the Indian subcontinent. Barolia and Sayani [17] reviewed possible risk factors for CVD in the Pakistani environment. Aziz et al. [18] observed 6.2% prevalence of heart attack (8.2% in women and 4.5% in men) for a suburban area of Karachi, the biggest city of Pakistan.

The males are more exposed to CVD-related deaths than females world-wide [3]. A number of studies are available in the literature regarding the gender-specific risk factors for CVD, and differences between the survival rates of male and female patients suffering from CVD. However, such literature in the Pakistani context is very rare. There is still a strong need to explore the gender-specific risk factors in Pakistan. This paper is an effort in this direction. The main objective of the present paper is to identify the informative risk factors for males and females having left ventricular systolic dysfunction of New York Heart Association (NYHA) class III and IV, and build gender-specific survival models for the survival predictions. This study considers the data (taken from [19]) comprising 299 heart failure patients of ≥40 years age who were admitted in one of the two main hospitals of Faisalabad, the third most populous city of Pakistan.

The remaining paper is arranged as follows. Section 2 describes the characteristics of the patients involved in the study, the procedure for collecting the information about risk factors, pre-processing of the data for the analysis, and the statistical techniques used. The discussion about the results of the analysis is given in Section 3. In Section 4, concluding remarks are given with some future recommendations.

## 2 Methods

### 2.1 Subjects

The data was collected from two main hospitals of Faisalabad, to build the gender-specific survival prediction models. The data consists of 299 patients (194 males and 105 females) of left ventricular systolic dysfunction of NYHA class III and IV. All the patients were of age 40 years or above and were admitted to Faisalabad Institute of Cardiology or Allied Hospital Faisalabad during the period April-December 2015. The average follow-up time was 130 days with a minimum of 4 days and a maximum of 285 days.

### 2.2 Risk factors

We describe below the observed risk factors and the data collection procedure: the HF data considered in this study can be accessed through Ahmad et al. [19]. However, to keep this paper stand-alone, the same data is being given as the supporting information. The patients of left ventricular systolic dysfunction were identified on the basis of their cardiac echo reports and notes written by the physician. The risk factors on which information was collected include age, gender, Blood Pressure (BP), smoking, diabetes, Ejection Fraction (EF), serum creatinine, Creatinine Phosphokinase (CPK), serum sodium, platelets count (pl), and anemia.

### 2.3 Data preprocessing

The creatinine levels were observed on a continuous scale. In general, the normal creatinine levels for men and women range from 0.9 to 1.3 mg/dL, and 0.6 to 1.1 mg/dL, respectively. According to these limits, the values of creatinine were classified into two categories, i.e., within and outside the normal range. The platelets count was also divided into two categories, i.e., within the normal range (150000-450000) and outside the normal range. The patients are categorized according to the age groups: ≤45, 45 − 50, 50 − 55, 55 − 60 60 − 65, 65 − 70, and >70. The values of EF were divided into three categories as normal (50 − 75), borderline (40 − 49) and very low (<40). A very low EF value (<40%) may be an evidence of heart failure or cardiomyopathy, but a too high value (>75%) in general is not related to heart failure although it may cause hypertrophic cardiomyopathy. Only one patient had an EF value greater than 75 and was, therefore, excluded from the analysis. As a result, *N* = 298 (*N*_male_ = 194 and *N*_female_ = 104) subjects are considered for model building. In the available data, patients with less than 36 hematocrit level have been considered as anemic [20].

### 2.4 Statistical techniques

The descriptive statistics are computed in terms of range, mean and standard deviation for continuous predictors, and in terms of frequencies and percentages for categorical predictors. Cox regression or proportional hazards regression [21, 22] is one of the most popular techniques used to model time-to-event variable with one or more predictors. If **X**_*n*×*p*_ is a data matrix of *n* observations recorded for *p* predictors, and ***β*** = (*β*_1_, …, *β*_*p*_) is the parameter vector associated with the predictors *X*_1_, …, *X*_*p*_ respectively, the Cox proportional hazards regression model can be written as follows:

$$\begin{array}{c} h\left(t\right)=h_{0}\left(t\right)\cdot \exp\left(X\beta \right), \end{array}$$ (1)

where *h*(*t*) is the expected hazard at time *t*, *h*_0_(*t*) is the baseline hazard representing the hazard when all *p* predictors assume zero value. In this text we use Cox proportional hazard regression to fit a survival model for all patients, male patients, and female patients, with different risk factors. The Kaplan-Meier curves [23] are used to study the difference in survival times of both genders. Lasso (least absolute shrinkage and selection operator) is the most commonly used approach to perform variable selection with regularization [24]. In case of a factor variable, the selection should not focus on the parameters but on the variable with all of its categories [25, 26]. Since our time-to-event data also contains factor variables, we use group lasso for selecting the informative risk factors in the gender-specific Cox model [27, 28]. For all factor variables, the normal range category is considered as the reference category. The informative risk factors were identified for the overall data of all patients, as well as for male and female patients separately. In each setting, after selection of the informative risk factors, a Cox model is fitted with the selected informative risk factors. The goodness of fit of the selected models is tested using Likelihood Ratio (LR) test. The concordance index (also called C-index) is also computed as a measure of the predictive performance of the selected models. In particular, the C-index for a survival model is the weighted average of area under time-specific ROC (Receiver Operating Characteristic) curves, or alternatively we can call it time-dependent AUC (Area Under the Curve). All computations and the analysis was done with the statistical programming language R.

## 3 Results

**Fig 1:**
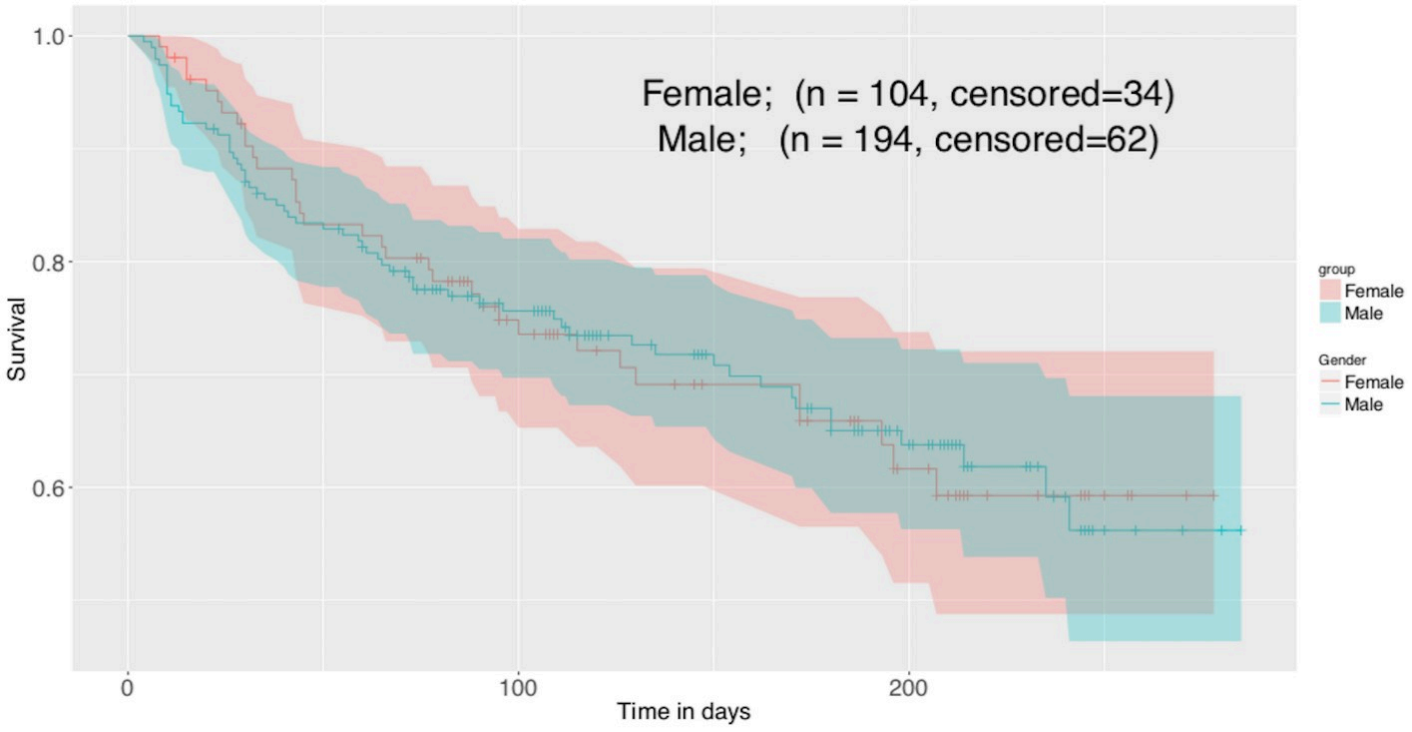
Kaplan Meier survival curves for males and females with 95% CI coverage.

**Fig 2:**
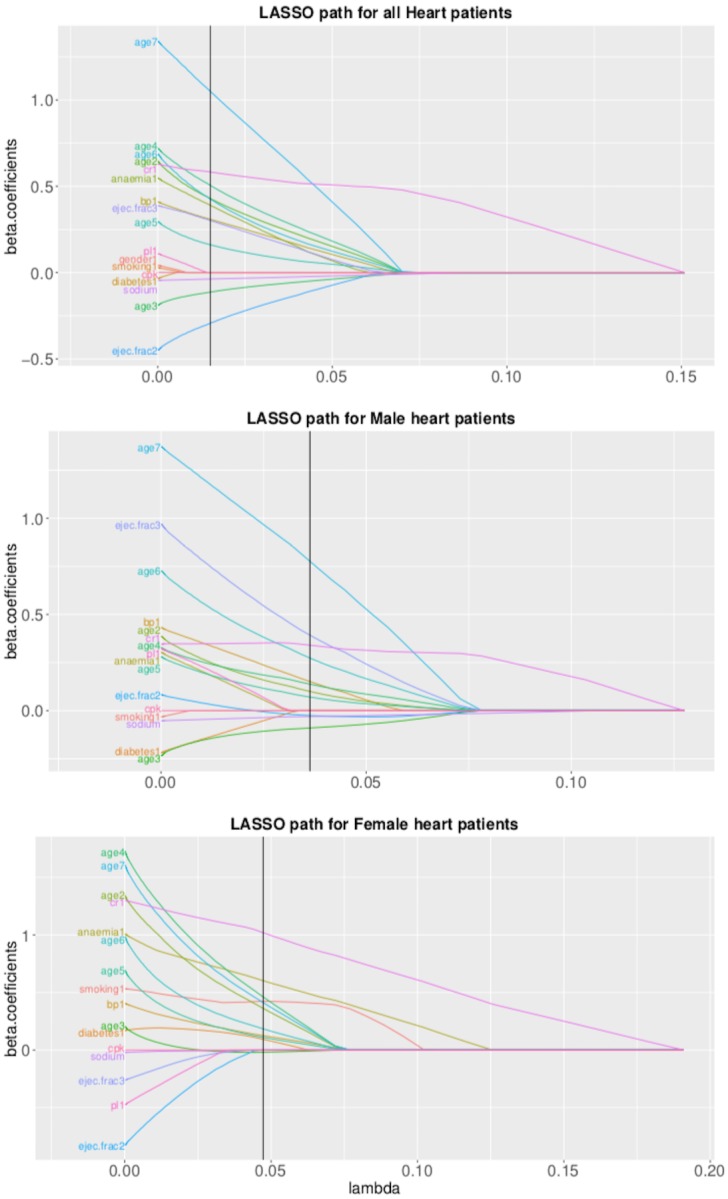
Lasso path for regression coefficients for overall heart data, data for males and females.

The descriptive statistics for different risk factors are given in Table 1. The Kaplan-Meier curves for males and females with 95% CI coverage are given in Fig 1. There are 34 censored observations for 104 female patients and 62 censored observations for 194 male patients. The Kaplan-Meier survival curves show a similar survival pattern for males and females. The group lasso recommended a different set of informative risk factors when applied to the whole data, males data and females data. The list of the selected risk factors in each case is given in Table 2. The risk factors selected with group lasso are marked with ✔, whereas the predictors that could not qualify (with zero regression coefficients) to be a part of the model are marked with ✘. The lasso path for the regression coefficients of overall heart data, males, and females is given in Fig 2. For the overall data, the risk factors gender, smoking, diabetes, BP, platelets count and CPK were not truly correlated with the patient’s survival. However, the situation changed when group lasso was applied to male and female data separately. In each case three risk factors, but different for both genders, were identified as non-informative. For male data, smoking, diabetes, and anaemia were not correlated with survival. For female data, however, EF, sodium and platelets count had zero regression coefficients. The basic assumption of proportional hazards should be fulfilled for fitting a Cox proportional hazards model. The violation of this assumption will lead to the biased estimates. The hypothesis test about the fulfillment of this assumption was tested before fitting Cox model with any number of predictors and the results are given in Table 3. The results show that the assumption of proportional hazards is fulfilled for all fitted models with p-value >.05. The results of the likelihood ratio test for testing the goodness of fit of the selected model as compared to the model with all observed risk factors are given in Table 4. The results show that the selection worked well and the selected models based on the informative predictors are better not only for whole data but also for gender-specific models. In order to assess the predictive ability of the selected models, the results of C-index and corresponding 95% CI for the models with selected risk factors and with all observed risk factors are also given in Table 4. The results show no difference in the index values for both selected and full model, indicating that the selected models are as good as the corresponding full models with respect to their predictive performance.

**Table 1:** Descriptive statistics for the observed risk factors. Mean and standard deviations are given for continuous risk factors. Frequencies and corresponding percentages are given for the categorical risk factors.

variable	levels	Total	Males	Females
gender		298	194	104
age				
age1	≤45	36(18.56)	25(12.89)	11(10.58)
age2	> 45 & ≤ 50	37(19.07)	18(9.28)	19(18.27)
age3	> 50 & ≤ 55	38(19.59)	28(14.43)	10(9.62)
age4	> 55 & ≤ 60	50(25.77)	32(16.49)	18(17.31)
age5	> 60 & ≤ 65	48(24.74)	29(14.95)	19(18.27)
age6	> 65 & ≤ 70	37(19.07)	22(11.34)	15(14.42)
age7	> 70	52(26.8)	40(20.62)	12(11.54)
EF				
EF1	> 50 & ≤ 75	59(30.41)	32(16.49)	27(25.96)
EF2	≥40 & ≤ 49	57(29.38)	37(19.07)	20(19.23)
EF3	<40 & > 75	182(93.81)	125(64.43)	57(54.81)
smoking	yes	96(49.48)	92(47.42)	4(3.85)
	no	202(104.12)	102(52.58)	100(96.15)
diabetes	yes	125(64.43)	70(36.08)	55(52.88)
	no	173(89.18)	124(63.92)	49(47.12)
BP	yes	105(54.12)	61(31.44)	44(42.31)
	no	193(99.48)	13(6.7)	60(57.69)
Anaemia	Yes	129(66.49)	77(39.69)	52(50)
	No	169(87.11)	117(60.31)	52(50)
cr	Yes	104(53.61)	57(29.38)	47(45.19)
	No	194(100)	137(70.62)	57(54.81)
pl	Yes	40(20.62)	29(14.95)	11(10.58)
	No	258(132.99)	165(85.05)	93(89.42)
		Mean ± SD (min, max)	Mean ± SD (min, max)	Mean ± SD (min, max)
sodium		136.62 ± 4.42 (113, 148)	136.54 ± 4.13 (113, 148)	136.79 ± 4.93 (116, 146)
CPK		581.84 ± 971.92 (23, 7861)	638.70 ± 1114.89 (23, 7861)	475.77 ± 614.24 (52, 3964)

**Table 2:** The selection of informative risk factors for overall data, and gender specific data using L1 penalization. The informative and non-informative variables are represented with a ✔ and ✘ respectively.

	gender	smoking	diabetes	BP	Anaemia	age	EF	sodium	cr	pl	CPK
Male & Female	✘	✘	✘	✔	✔	✔	✔	✔	✔	✘	✘
Male	-	✘	✘	✔	✘	✔	✔	✔	✔	✔	✔
Female	-	✔	✔	✔	✔	✔	✘	✘	✔	✘	✔

**Table 3:** Global proportional hazards assumption test results for full model with all observed risk factors, and selected model based on informative risk factors.

	*χ*^2^	p-value
Male & Female (Full Model)	14.67	0.62
Male & Female (Selected Model)	12.49	0.41
Male (Full Model)	12.05	0.74
Male (Selected Model)	11.29	0.59
Female (Full Model)	17.27	0.37
Female (Selected Model)	15.25	0.23

**Table 4:** Likelihood Ratio (LR) test results regarding goodness of fit of the selected model with respect to the full model with all observed risk factors. The results of C-index with corresponding 95% CI are given to examine the predictive performance of different models.

	LR Test		Concordance Coeff. C with 95% CI			
	*χ*^2^	p-value	Full Model		Selected Model	
			C	CI	C	CI
Male & Female	4.25	0.51	0.73	(0.67, 0.79)	0.72	(0.66, 0.78)
Male	1.81	0.61	0.74	(0.66, 0.82)	0.73	(0.66, 0.81)
Female	2.17	0.70	0.78	(0.68, 0.89)	0.77	(0.67, 0.88)

The hazard ratios with corresponding 95% CI for the selected models for all patients, and for male and female patients are given in Table 5. The risk factors BP, age, and creatinine were common in all models. The expected hazard relative to one mmHg increase in BP is higher in males than females. The hazard of creatinine outside the normal range (given in Section 2.3) is more sever in females than males. The risk of death with abnormal creatinine is 4.45 times (for females) and 1.47 times (for males) as compared to the corresponding group with normal level of creatinine. The males older than 70 years have maximum risk of death whereas the females of age group 55-60 have the highest risk of death as compared to the the reference age group i.e., ≤45 years. In our study, the anaemia was a risk factor associated with females, and the females suffering from anaemia were 2.79 times more exposed to the hazard than those who were not suffering from this problem. A low Ejection Fraction (<40%) is a risk of death in male patients and such males have 2.5 times higher risk of hazard than those whose Ejection Fraction is in normal range (50-75). In the lasso regression, the coefficient of CPK (Creatinine Phosphokinase) was very close to zero i.e., 4.11e-05 and 1.57e-04 for males and females respectively. The inclusion of CPK with such small coefficient reflected equal hazard rate (for males and females) in normal range group and the group having CPK outside normal range. In this study, smoking and diabetes were not informative risk factors for males. The smoker females have 1.43 times higher risk of death than non-smoker females. Also, the chances of death are 41% higher in diabetic females than those who do not have diabetes problem. The platelets count was an informative risk factor for predicting the survival in males only. The males having this count outside the normal range are 36% more exposed to the hazard than their counterparts with normal range.

**Table 5:** Hazard Ratios for informative risk factos with 95% Confidence Interval (CI) for all patients, and male and female patients.

	Male & Female		Male		Female	
	HR	95% CI	HR	95% CI	HR	95% CI
smoking	-	-	-	-	1.43	(0.38, 5.35)
diabetes	-	-	-	-	1.41	(0.66, 3.01)
BP	1.45	(0.95, 2.21)	1.55	(0.90, 2.68)	1.37	(0.67, 2.81)
anaemia	1.58	(1.04, 2.41)	-	-	2.79	(1.22, 6.37)
age2	1.79	(0.69, 4.62)	1.55	(0.42, 5.74)	3.91	(0.47, 32.45)
age3	0.82	(0.27, 2.46)	0.87	(0.24, 3.14)	1.22	(0.11, 13.82)
age4	1.92	(0.79, 4.66)	1.44	(0.51, 4.09)	5.27	(0.65, 42.69)
age5	1.17	(0.44, 3.11)	1.38	(0.45, 4.22)	1.96	(0.20, 19.28)
age6	1.76	(0.68, 4.57)	2.15	(0.70, 6.66)	2.77	(0.30, 25.37)
age7	3.74	(1.61, 8.70)	4.39	(1.74, 11.07)	4.62	(0.55, 39.04)
EF2	0.66	(0.28, 1.54)	1.09	(0.32, 3.72)	-	-
EF3	1.57	(0.87, 2.83)	2.50	(0.96, 6.53)	-	-
sodium	0.96	(0.92, 1.00)	0.95	(0.90, 1.01)	-	-
cr	1.74	(1.11, 2.73)	1.47	(0.81, 2.66)	4.45	(1.90, 10.40)
pl	-	-	1.36	(0.69, 2.69)	-	-
CPK	-	-	1.00	(1.00, 1.00)	1.00	(1.00, 1.00)

## 4 Conclusion

The characteristics, medical history, and treatment of male and female HF patients can be different [7]. Also, in Pakistani society, women are facing worse quality of life than men with the same severity of HF. Our study shows almost similar survival pattern in males and females. However, in literature, many studies concluded women as better survivors than men with HF [13, 29–31]. According to our findings, gender as a risk factor is not truly correlated with the survival of a patient. Furthermore, this study explores a different set of informative risk factors for males and females HF patients to predict their survival. Our findings are consistent with some other studies [6, 32]. It is possible to question the generalizability of our findings because the data is collected from only two main hospitals of Faisalabad. But it should also be kept in mind that 14.177 million population (51.01% males and 48.98% females) of Faisalabad division (comprising four districts) in general, and 7.874 million population (51.2% males and 48.8% females) of Faisalabad district in particular, is depending on only three main hospitals for CVD treatment facilities. The risk factors considered in this study are based on cardiac echo reports or the physician notes. Some other unmeasured variables could confound the results. Another limitation of the study is that all the patients of age ≥40 were considered, which excluded the younger women with pregnancy. In sum, a well designed prospective study, with sufficient sample size and without under-representation of any gender, in Pakistani setup is still needed to verify our findings and to identify the gender-specific risk factors for predicting the survival among the male and female HF patients.

## Supporting information

S1 File The data used in this paper. (CSV)
